# Two‐year outcomes of minimally invasive XEN Gel Stent implantation in primary open‐angle and pseudoexfoliation glaucoma

**DOI:** 10.1111/aos.14627

**Published:** 2020-09-30

**Authors:** Teresa Rauchegger, Reinhard Angermann, Peter Willeit, Eduard Schmid, Barbara Teuchner

**Affiliations:** ^1^ Department of Ophthalmology and Optometry Medical University of Innsbruck Innsbruck Austria; ^2^ Department of Neurology Medical University of Innsbruck Innsbruck Austria; ^3^ Department of Public Health and Primary Care University of Cambridge Cambridge UK

**Keywords:** Ab interno, glaucoma, minimally invasive glaucoma surgery, XEN Gel Stent

## Abstract

**Purpose:**

The aim of this study was to evaluate the efficacy of XEN^®^ Gel Stent implantation in the treatment of primary open‐angle glaucoma (POAG) and pseudoexfoliation glaucoma (XFG) regarding the reduction of intraocular pressure (IOP) and number of IOP‐lowering medications over 2 years.

**Methods:**

In this retrospective, observational, single‐centre study, patients with POAG or XFG underwent implantation of the XEN^®^ Gel Stent with or without combined phacoemulsification. Changes in mean IOP, mean number of IOP‐lowering medications, number of postoperative interventions, complete or qualified surgical success rate (defined as IOP < 18 mmHg without or with IOP‐lowering medication, respectively) and complete surgical failure rate (defined as the necessity of a glaucoma‐related secondary surgical intervention or loss of light perception) were assessed 12 months (12M) and 24 months (24M) postoperatively.

**Results:**

Seventy‐nine eyes of 63 patients with open‐angle glaucoma were included in the study (71% POAG, 29% XFG). Before surgery, mean IOP was 23.4 ± 7.9 mmHg. IOP was 14.6 ± 3.6 mmHg 12 months postoperatively (−31% from baseline, 95% CI −42% to −20%, *n* = 30, p < 0.001) and 14.8 ± 4.4 mmHg 24 months postoperatively (−29% from baseline, 95% CI −30% to −41%, *n* = 28, p < 0.001). Mean number of IOP‐lowering medications was significantly reduced from 2.7 ± 1.1 before surgery to 1.0 ± 1.2 (−69%, 95% CI −89% to 46%, p < 0.001) 12 months after surgery and 1.0 ± 1.2 (−64%, 95% CI −91% to −36%, p < 0.001) at 24 months after surgery. Complete surgical success was achieved in 39% (12M) and 34% (24M) of patients and qualified success in 29% (12M) and 27% (24M). 13 (16%) eyes were classified as complete surgical failure. In 62% of the patients needling procedures had to be performed.

**Conclusion:**

The XEN^®^ Gel Stent is an efficacious minimal invasive glaucoma surgery for primary open‐angle and pseudoexfoliation glaucoma, resulting in significant reduction of IOP and a reduction in glaucoma medications from baseline in two‐third of treated patients with 2‐year follow‐up. Frequent follow‐up examinations were mandatory to identify early and late bleb failure and additional needling procedures were necessary to reestablish aqueous flow.

## Introduction

Glaucoma is the second leading cause of blindness and the leading cause of irreversible blindness worldwide (The International Agency for the Prevention of Blindness 2019). Current treatment of glaucoma is mainly based on lowering intraocular pressure (IOP) to an individual target level in order to prevent or decelerate glaucoma progression and blindness. Glaucoma surgery is necessary when medical therapy does not provide adequate IOP reduction. Trabeculectomy is the most commonly performed incisional glaucoma procedure and is considered the gold standard. Although highly effective, the possibility of vision‐threatening complications of trabeculectomy has led to the development of less invasive glaucoma procedures, collectively called MIGS (minimally invasive glaucoma surgery), lowering the IOP without extensive surgical dissection (Sheybani 2015; Bar‐David & Blumenthal 2018).

One new development of MIGS is the XEN^®^ Gel Stent (XEN^®^ 45; Allergan Plc., Dublin, Ireland). The stent is a hydrophilic tube, made of porcine gelatin cross‐linked with glutaraldehyde, with a length of 6 mm and an inner lumen of 45 µm. It is inserted through a clear cornea incision and placed in the subconjunctival space at the superonasal quadrant using a preloaded injector. XEN^®^ 45, the only one currently available on European markets, lowers IOP by shunting aqueous humour from the anterior chamber to the subconjunctival and subtenon space bypassing the trabecular meshwork. This offers the opportunity to increase aqueous humour outflow by a minimally invasive procedure creating a filtering bleb without surgical opening of the conjunctiva (Lewis 2014; Sheybani et al. 2015).

The aim of this study was to evaluate the efficacy of XEN^®^ Gel Stent as a minimally invasive intervention in reducing the IOP and IOP‐lowering medications over 2 years.

## Patients and Methods

We performed a single‐centre, retrospective study of patients who underwent implantation of XEN^®^ Gel Stent with or without combined phacoemulsification between 2016 and 2018. Patients with primary open‐angle glaucoma (POAG) or pseudoexfoliation glaucoma (XFG) with medically uncontrollable IOP or intolerance of topical therapy were included in this study. Patients with other types of glaucoma were excluded. After complete preoperative ophthalmic examination, postoperative evaluations were performed at 1 day, 1 week and then every month up to 2 years. At baseline, demographic data, prior surgical procedures, type of glaucoma, measurement of IOP with Goldmann applanation tonometry, best corrected visual acuity in Snellen (BCVA) and visual field (VF) mean deviation in Octopus perimeter (Haag Streit International, Koeniz, Switzerland) were collected. The postoperative collected data included IOP readings, number of glaucoma medication, BCVA, number of needling procedure and if applicable type of secondary IOP‐lowering procedure. After obtaining patient’s informed consent, data were also collected from private practice ophthalmologists postoperatively.

### Surgical technique

Surgery was performed under local anaesthesia. After insertion of the eyelid retractor, the target sector was marked in the nasal upper quadrant 3–4 mm from the limbus. Then 0.1 ml of diluted Mitomycin C (0.1 mg/ml) was injected subconjunctivally adjacent to the target sector. Two corneal paracenteses were created and a viscoelastic substance (Healon Pro; Johnson & Johnson Vision, Santa Ana, CA, USA) was injected into the anterior chamber. The 27‐gauge preloaded injector (XEN^®^ 45; Allergan Plc.) was inserted through the paracentesis at the inferotemporal quadrant. The eye was stabilized with a Vera Hook through the second nasal paracentesis. The needle was then directed across the anterior chamber and the stent was implanted at the superonasal part of the anterior chamber angle. As the bevel of the needle was visible under the conjunctiva in the previously marked area, the injector was rotated and the XEN^®^ Gel Stent was injected in the subconjunctival space. The ideal placement of for the stent would be 1 mm in the anterior chamber, 2 mm in the sclera and 3 mm in the subconjunctival space. The mobility of the subconjunctival part of the stent was checked by moving the end of the implant slightly from side to side under the conjunctiva. The position of the inner part of XEN^®^ Gel Stent was checked by gonioscopy. The viscoelastic was removed from the anterior chamber and irrigation with balanced salt solution (BSS) was used to check the implant function and the formation of the bleb. The corneal incisions were hydrated with balanced salt solution.

In the combined cataract group the XEN^®^ Gel Stent was implanted, as above described, after a routine phacoemulsification with intraocular lens implantation.

On the day of surgery, all local and systemic antiglaucomatous medications were stopped. Postoperatively, anti‐inflammatory eye drops (Prednisolone acetate 1% or equivalent) hourly, slowly reduced over several months depending on the bleb appearance, and antibiotics (Ofloxacin or equivalent) four times a day for a week were applied.

Postoperative needling was performed under local topical anaesthesia by sweeping a 27‐gauge needle in the subconjunctival space around the XEN^®^ implant in order to dissect subconjunctival and subtenon scarring. At the end of the procedure, a volume of 0.05 ml of diluted MMC solution (0.1 mg/ml) was injected in the newly formed subtenon space.

### Statistical analysis

The primary efficacy outcomes of the study were mean IOP and reduction of the mean number of medications between baseline and 12 or 24 months after XEN^®^ Gel Stent implantation, respectively. In accordance to the guidelines of the World Glaucoma Association (Shaarawy et al. 2009), surgical success was defined as postoperative IOP ≤ 18 mmHg and ≥20% IOP reduction without (complete success) or with ocular hypertensive medication (qualified success) without a secondary IOP‐lowering procedure.

Complete surgical failure was defined as the necessity of further glaucoma‐related secondary surgical intervention (with the exception of needling procedure) or loss of light perception. The mismatch of above defined IOP criteria was not defined as a complete surgical failure because a single elevation IOP might also be treated conservatively.

Needling was documented as an additional postoperative treatment to improve aqueous flow and lower IOP.

Categorical variables are summarized as numbers of patients and percentages, while continuous variables are summarized as means and 95% confidence intervals. Percentage changes were calculated by comparison to levels before surgery. The Kolmogorov–Smirnov test was employed to test variables for normal distribution. *t*‐Tests and analysis of variance for normally distributed data were employed. Categorical data were compared using a chi‐squared test and Fisher’s exact test. All p‐values ≤0.05 were considered significant. All statistical analyses in this report were performed by ibm spss Statistics 25 (IBM, Armonk, NY, USA).

## Results

Patient demographics and baseline characteristics are shown in Table 1.

**Table 1:** Patients baseline characteristics and demographics.

Demographics/characteristics	Singe procedure	Combined with Phaco	Total
	*N* = 56	*N* = 23	*N* = 79
Sex, *n* (%)			
Female	34 (69.4)	15 (30.6)	49 (62)
Male	22 (73.3)	8 (26.7)	30 (38)
Glaucoma type, *n* (%)			
Primary open angle	39 (69.6)	17 (30.4)	56 (70.9)
Secondary open angle	17 (73.9)	6 (26.1)	23 (29.1)
Previous glaucoma procedures, *n* (%)			
Trabeculectomy	12 (80.0)	3 (20.0)	15 (19)
Transscleral cyclophotocoagulation	2 (100)	0	2 (2.5)

IOP = intraocular pressure; Phaco = phacoemulsification with intraocular lens implantation; SD = standard deviation.

Between 2016 and 2018 a total of 101 XEN^®^ implantations were performed. Seventy‐nine eyes of 63 patients met inclusion criteria. Fifty‐six (71%) were primary open‐angle glaucoma (POAG) and 23 (29%) were pseudoexfoliation glaucoma (XFG). Forty‐nine (62%) women and 30 (38%) men were included. In 23 eyes (29%), XEN^®^ implantation was combined with cataract surgery, while 56 eyes (71%) were treated in a single procedure. A total of 45 (57%) eyes were already pseudophakic in the single procedure group. Thirteen (16%) out of 79 eyes needed a glaucoma‐related secondary surgical intervention and were classified as complete surgical failure. One patient died during the study period (unrelated to the device), who had both eyes implanted. Four patients were lost to follow‐up. Therefore, 60 out of 79 eyes were analysed for IOP change and change in number of IOP‐lowering medication. Overall, 30 eyes completed the 12‐month visit and 28 eyes the 24‐month visit.

### Intraocular pressure

**Fig. 1:**
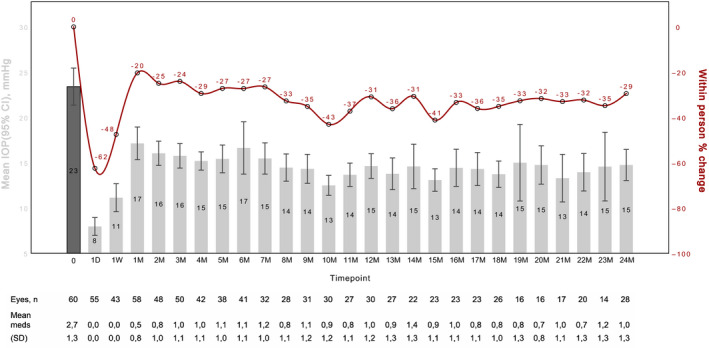
Mean change in intraocular pressure (IOP) and number of IOP‐lowering medication from preoperative baseline over time.

Mean ± SD IOP preoperatively was 23.4 ± 7.9 mmHg. It was significantly reduced to 14.6 ± 3.6 mmHg at 12 months (−31%, 95% CI −42% to −20%, *n* = 30, p < 0.001) and 14.8 ± 4.4 at 24 months (−29%, 95% CI −30% to −41%, *n* = 28, p < 0.001) (Fig. 1).

The outcomes were similar in the single procedure group versus the combined cataract surgery group in the first 12 months: mean IOP change was −30% (95% CI −42% to −18%) in the single procedure group versus −37% (95% CI −58% to −16%) in the combined cataract group (p value for difference p = 0.25). The IOP change after 24 months appear somewhat lower in the combined cataract group [−15% (95% CI −83% to 53%) versus −32% (95% CI −43% to −21%) in the single procedure group], but this was statistically non‐significant (p value for difference p = 0.18).

There was no significant difference in mean IOP reduction between the POAG and XFG group at both endpoints [12 months: POAG −29% (95% CI −40% to −17%), XFG −39% (95% CI −69% to −9%), p = 0.96]; 24 months: POAG −22% (95% CI −36% to −9%), XFG −51% (95% CI −63% to 39%, p = 0.1).

### Number of intraocular pressure‐lowering medication

Mean ± SD number of medications decreased significantly from 2.7 ± 1.1 preoperatively to 1.0 ± 1.2 at 12 months and 1.0 ± 1.2 at 24 months. Mean change of IOP‐lowering medications was −69% (*n* = 29, 95% CI −89% to 46%, p < 0.001) at 12 months and −64% at 24 months postoperatively (*n* = 27, 95% CI −91% to −36%, p < 0.001) (Fig. 1).

Regarding the mean number of medications, there was no statistical difference between the solo procedure and the combined cataract surgery group. In the solo procedure group mean number of medications decreased −72% (95% CI −90% to 55%) at 12M and −66% (95% CI −90% to −42%) at 24M. In the combined cataract group mean number of medications decreased −44% (95% CI −170% to 82%) at 12M and −50% (95% CI −115% to 14%) at 24M (p value for difference p = 0.96 12M and p = 0.28 at 24M).

The outcomes also appeared similar between patients with primary open‐angle and pseudoexfoliation glaucoma (12 months: POAG −66% (95% CI −87% to −45%), XFG −80% (95% CI −112% to –48%), p = 0.2); 24 months: POAG −55% (95% CI −82% to −28%), XFG −89% (95% CI −116% to 63%, p = 0.07).

### Surgical success

Thirty‐nine per cent achieved complete surgical success at 12 months and 34% at 24 months. Qualified success was achieved in 29% at 12 months and 27% at 24 months.

Subgroup analysis showed complete surgical success in 40% (POAG) versus 40% (XFG) at 12M and 27% (POAG) versus 55% (XFG) at 24M. A qualified surgical success was achieved in 36% (POAG) versus 30% (XFG) at 12M and 33% (POAG) and 9% (XFG) at 24M.

### Needling procedures

**Fig. 2:**
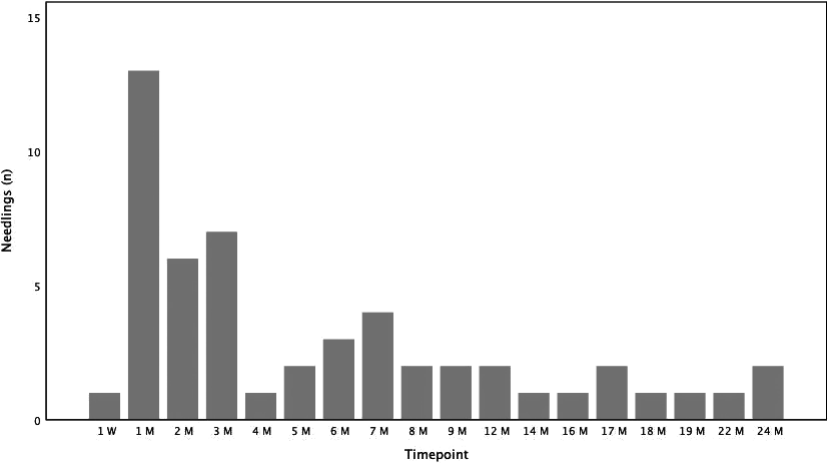
Number of needling procedures over 24 months.

Thirty‐seven (62%) patients required a needling procedure. All in all, 52 needling procedures were performed. In the majority of cases a needling was necessary within the first month postoperatively (25%) (Fig. 2). Most of the needled eyes (*n* = 25, 42%) needed one procedure, 9 (15%) eyes received two needling procedures and three patients (5%) three needling procedures.

The number of needling procedures was evenly distributed in each group.

In the single procedure group 64% of the patients needed at least one needling and in the combined cataract group 56%. In patients with POAG, a needling procedure was performed in 62% versus 61% in patients with XGF. Detailed information of needling procedures is displayed in Table 2.

**Table 2:** Comparison of needling procedures in each group.

Group	Patients requiring needling procedure (% per group)		
	1×	2×	3×
Singe procedure	18 (41)	7 (16)	3 (7)
Combined cataract	7 (44)	2 (13)	0
POAG	17 (41)	6 (14)	3 (7)
XFG	8 (44)	3 (17)	0

### Complete surgical failure

**Fig. 3:**
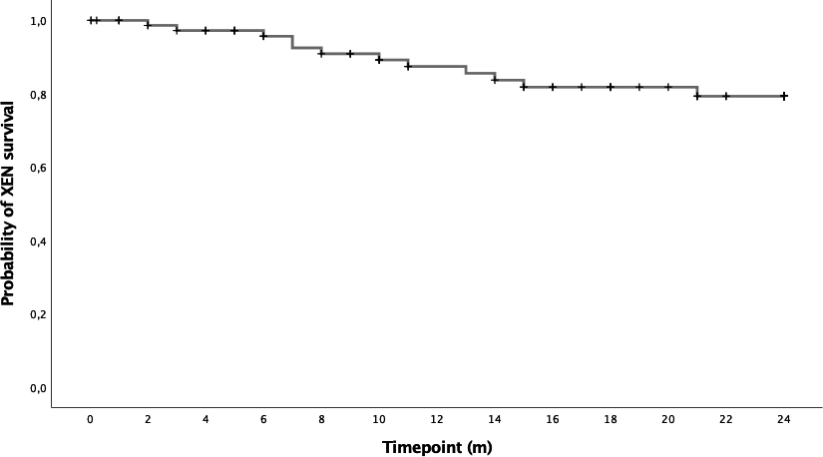
Kaplan–Meier curve showing the probability of XEN^®^ survival.

Thirteen (16%) out of 79 eyes needed a glaucoma‐related secondary surgical intervention and were therefore classified as complete surgical failure. Nine (11%) received a trabeculectomy and 4 (5%) a second XEN^®^ implantation. No patient suffered from a loss of light perception. Figure 3 shows the Kaplan–Meier curve of the probability of XEN^®^ survival during 24 months.

Postoperative Interventions and glaucoma‐related secondary surgical intervention are listed in Table 3.

**Table 3:** Postoperative Interventions and glaucoma‐related secondary surgical intervention

Interventions	Number of eyes (%)
Needling procedures	37 (62)
1×	25 (42)
2×	9 (15)
3×	3 (5)
Secondary surgical intervention	14 (18)
Trabeculectomy	10 (13)
Second XEN implantation	4 (5)

### Safety

Regarding the safety profile, the incidence of ocular adverse events is displayed in Table 4.

**Table 4:** Ocular adverse events reported throughout the 2‐year study

Adverse events	Number of eyes (%)
Hyphema*	5 (3.9)
Hypotony with choroidal effusion^†^	3 (2.4)
Shallow anterior chamber	1 (0.8)
Glaucoma attack	1 (0.8)
Stent dislocation	1 (0.8)
Stent fracture	1 (0.8)
Total	12 (9.5)

* Self‐resolved, lasting <7 days.

^†^ Self‐limiting, lasting <60 days.

In 12 (9.5%) out of 79 patients, an ocular adverse event was documented. The most frequent reported postoperative complications were hyphema (*n* = 5, 3.9%), self‐resolving within 7 days postoperatively, and hypotony with choroidal effusion (*n* = 3, 2.4%), self‐limited within the first two postoperative months. One patient had a shallow anterior chamber, resolving within the first postoperative week. A further patient developed a glaucoma attack with an IOP >50 mmHg at month 6 months. One XEN^®^ Gel Stent was dislocated into the subconjunctival space and one XEN^®^ Gel Stent was fractured, all of which occurred within the first month postimplantation.

## Discussion

The results of this current study suggest that the XEN^®^ Gel Stent significantly reduces IOP and number of IOP‐lowering medications. While other studies report 12 months results, our study shows long‐term efficacy of XEN^®^ Gel Stent implantation over a time period of 24 months.

We observed a significant drop of IOP from 23.4 ± 7.9 mmHg preoperatively to 14.6 ± 3.6 mmHg at month 12 and 14.8 ± 4.4 mmHg at month 24 postoperatively. We found a mean IOP reduction from baseline of −30.9% at month 12 and −29.4% at the end of the follow‐up at month 24. The mean IOP‐lowering medication decreased from 2.7 ± 1.1 at baseline to 1.0 ± 1.2 at month 12 and 1.0 ± 1.2 at month 24. In addition, 34% of the patients attaint target IOP without any medication after 24 months.

These results are comparable with the prospective study by Reitsamer et al. (2019) who showed a reduction of IOP from 21.4 ± 3.6 mmHg (medicated baseline) to 14.9 ± 4.5 mmHg at month 12 and 15.2 ± 4.2 mmHg at month 24. Gabbay et al. (2019) reported, that IOP was reduced from 22.1 ± 6.5 mmHg to 15.4 ± 5.9 after 12 months and 14.5 ± 3.3 after 24 months in a retrospective, non‐comparative audit of patient records in the UK. In addition, similar outcomes were noted in studies with 1‐year follow‐up. Grover et al. (2017) showed in a multicentred study including 65 patients with OAG that the mean IOP decreased after XEN45 Gel Stent implantation from 25.1 ± 3.7 mmHg to 15.9 ± 5.2 mmHg 12 months postoperatively. Galal et al. (2017) reported in a smaller study population of 13 eyes an IOP drop from 16 ± 4 mmHg pre‐op to 12 ± 3 mmHg 1 year postsurgery.

Absolute mean postoperative IOP values after trabeculectomy reported in the literature are between 11.0 and 15.5 mmHg (Lochhead et al. 2003; Al‐Haddad et al. 2017). The mean postoperative IOPs after XEN^®^ Gel Stent are therefore comparable to trabeculectomy results. Marcos Parra et al., who compared XEN stent with trabeculectomy, found that the XEN implant significantly reduces IOP to a similar rate than trabeculectomy. The mean postoperative IOP reduction after trabeculectomy reported in this study was 33.8% (Marcos Parra et al. 2019). Compared to that, we achieve a similar postoperative IOP reduction by implanting the XEN^®^ Gel Stent (−30.9% 12M and −29.4% 24M). Our results are consistent with those published by Reitsamer et al. (−29.3% reduction after 12 months, −27.8% after 24 months) and Grover et al. (−35.6% reduction after 12 months) (Grover et al. 2017; Reitsamer et al. 2019).

The reduction of antiglaucoma medications of −69% (12M) and −65% (24M) in our study is comparable to results reported by Gedde et al. (2012), who described a reduction −59% after trabeculectomy.

Previous studies have suggested that a combined cataract surgery with trabeculectomy may be less successful in reducing IOP than trabeculectomy alone (Friedman et al. 2002; Lochhead et al. 2003). Our results show no such difference in the single procedure group vs the combined XEN^®^ implantation with cataract surgery. Tallying with our results, Hohberger et al. (2018) reported in a large cohort of 111 patients no significant difference between single XEN^®^ Gel Stent implantation and combined XEN^®^ Gel stent implantation and cataract surgery.

Pseudoexfoliation and pigment dispersion are known risk factors for rapid glaucoma disease progression (Chan et al. 2017). We compared the results between eyes with primary and secondary open‐angle glaucoma. Our data show no significant difference regarding IOP reduction in these two groups. These results match the data published by Gillmann et al. (2019), who reported in a prospective, interventional study a 26.8% reduction of IOP in patients with POAG and 28.3% IOP reduction in patients with XFG after 24 months. In comparison, Hohberger et al. (2018) showed a reduction of IOP <18 mmHg without any medications in patients with XFG in 60.0% after combined XEN^®^ implantation with cataract surgery in comparison of 46.7% in patients with POAG. In the stand‐alone group 25.9% of the patients with XFG and 57.4% of the patients with POAG achieved these results after 12 months.

In this study, we found a failure rate of 17% after 2 years. After trabeculectomy, a higher rate of failure has been reported in literature. In the tube versus trabeculectomy study, a failure rate of 28.2% after trabeculectomy was observed at 2 years (Gedde et al. 2009).

A notable factor is the large percentage of needlings performed. More than 60% of the patients needed at least one needling within 2 years. These results differ from the studies by Reitsamer et al. and Gabbay et al. who described an overall needling rate of only 42.1% and 36.8% after 2 years follow‐up, respectively (Gabbay et al. 2019; Reitsamer et al. 2019). In contrast, a study by Tan et al. (2018) showed a high needling rate with 51.3% after 1 year, which is more consistent with our results. If an encapsulation of bleb caused by aqueous humour is likely or fibrosis occurs less then 3 weeks postoperatively, recommendations for postoperative management of XEN^®^ Gel Stent include the use of preservative‐free aqueous suppressant with reevaluation of the bleb function at a later date as a reasonable first approach besides needling (Vera et al. 2019). Other studies suggest that surgeons should not wait too long to intervene by needling if IOP is too high in the early postoperative period, because increased IOP stretches the bleb, which incites fibroblast proliferation and fibrosis. A vicious cycle should be interrupted at the earliest to prevent failure of the surgery (Wang et al. 2007; Midha et al. 2019). In our experience administering aqueous suppressant does not resolve bleb dysfunction sufficiently in the early postoperative phase and we therefore decided upon an early needling strategy. This could be the reason why our needling rates are higher than those in other studies with a 2‐year follow‐up.

In our study, the number of needling procedures was evenly distributed in patients with POAG and XFG. In patients with POAG, a needling procedure was performed in 62% versus 61% in patients with XGF. Gillmann et al. (2019) showed a similar distribution between these groups. By 24 months needling was performed in 42.8% in patients with POAG and 43.2% in patients with XFG.

The highest risk for receiving a needling was within the first month, but needling procedures had to be performed over the entire observational period.

Although needling procedures are an effective postoperative intervention to restore bleb function, patients have to be frequently checked for early and late signs of bleb failure.

The XEN^®^ Gel Stent exhibits a good safety profile. Regarding this, the results of this study are like those previously published (Galal et al. 2017; Lenzhofer et al. 2018; Mansouri et al. 2018; Marcos Parra et al. 2019). The most common complication was a postoperative hyphema (3.9%), similar to what was reported by Marcos Parra et al. (2019). All cases of hypotony with choroidal effusion (2.4%) were self‐limited and self‐resolved within the first two postoperative months. These results did not differ from those reported by Reitsamer et al. (2019). The low incidence of complications after implantation of XEN^®^ Gel Stent may lead to a better quality of life (Hirooka et al. 2017; Marcos Parra et al. 2019).

There are several limitations to this study. While our study well reflects XEN implementation in real‐life conditions, it is – as a retrospective study – prone to selection and information biases. Furthermore, only a subset of participants had information on the primary endpoint assessed after 2 years of follow‐up (i.e. 28 out of 60 patients enrolled at baseline). This is primarily because patients are often followed up by private practice ophthalmologists, who referred patients to our clinic only if IOP was not manageable. Despite these reductions in sample sizes over follow‐up, we were able to demonstrate significant results throughout the entire observational period.

As we included both solo and combined cataract procedures, results may include some IOP reduction related to phacoemulsification. Intraocular pressure (IOP) change after 24 months appeared somewhat lower in the combined cataract group, yet this was not statistically significant. As subgroups were small in sizes, some differences may not lead to statistically significant results. On the other hand, previous studies show that cataract extraction in patients with uncontrolled glaucoma alone results in only mild IOP‐lowering effects with significant risk of IOP spikes and more aggressive treatment to control IOP postoperatively (Slabaugh et al. 2014a; Slabaugh et al. 2014b). Finally, this study was conducted in a single‐centre setting. This may decrease the generalizability of the results, but could also be regarded as a strength. All surgical indications and XEN^®^ implantations were performed by the same two surgeons, making our treatment regime very consistent despite the retrospective design.

In conclusion, this study demonstrates that the XEN^®^ Gel Stent is an efficacious minimal invasive glaucoma surgery procedure for primary open‐angle and pseudoexfoliation glaucoma, leading to significant lower IOP values and a reduction in glaucoma medications from baseline for at least 2 years. Frequent follow‐up examinations to recognize early and late bleb failure and additional needling procedures to improve aqueous flow and lower IOP are necessary.
